# Nurses’ perspectives about communication with patients in an intensive care setting using a communication board: A pilot study

**DOI:** 10.4102/hsag.v24i0.1162

**Published:** 2019-07-25

**Authors:** Martelize Gropp, Ensa Johnson, Juan Bornman, Rajinder Koul

**Affiliations:** 1 Centre for Augmentative and Alternative Communication, University of Pretoria, Pretoria, South Africa; 2 Department of Communication Sciences and Disorders, Moody College of Communication, The University of Texas at Austin, Austin, United States

**Keywords:** augmentative and alternative communication, communication board, intensive care setting, nurses, patients, vulnerable communicators

## Abstract

**Background:**

Communication in the intensive care setting (ICS) is critical for both the patient and the medical staff to provide efficient care and thus alleviate possible patient adverse effects. Persons with complex communication needs are particularly vulnerable in ICSs and therefore require additional communication support.

**Aim:**

This study focused on the perspectives of nurses about communication with patients with communication needs in ICSs using paper-based communication boards, namely the translated Vidatak EZ Board, before and after a training session.

**Setting:**

A 1650-bed public hospital with a 26-bed ICS in a semi-urban, low socio-economic area in South Africa served as the research setting.

**Methods:**

A quasi-experimental pre-test post-test group design with withdrawal and a control group was used. Data were gathered using a custom-designed questionnaire completed by ICS nurse participants recruited from a public hospital.

**Results:**

Responses of some nurses did not change in post-test 1, but their responses did change in post-test 2. Some of the nurses’ perspectives changed, as expected from the pre-test to post-test 1. Nurses recommended specific adaptations to the communication board.

**Conclusions:**

Most nurses agreed that communication is crucial in ICSs and that a communication board can be implemented; however, limited success was observed implementing the board following a short training. The inter-professional collaboration between nurses and speech-language therapists to provide optimal health care to patients in ICS is emphasised.

## Introduction

Patients in intensive care settings (ICSs) often have an endotracheal tube or a tracheostomy tube inserted to assist with respiration which affects their spoken language (Garrett et al. 2007; Handberg & Voss 2018; Martinho & Rodrigues 2016). Those patients who communicate using hand gestures and written language may equally be affected if their hands are restrained or if they have an intravenous drip inserted (Garrett et al. 2007; Handberg & Voss 2018; Happ et al. 2014, 2015). Because of these difficulties, communication between patients and nurses may be ineffective and frequent communication breakdowns may occur resulting in an increased risk to patients’ quality of care and well-being (Costello 2000; Ten Hoorn et al. 2016; Martinho & Rodrigues 2016; Patak et al. 2009). Apart from patients’ vulnerability, ICS nurses also experience frustration in their care for patients with communication difficulties, because they have insufficient information to address patients’ needs (Grossbach, Stranberg & Chlan 2010).

Not only is the need for effective communication between nurses and patients imperative to evade adverse medical events (Finke, Light & Kitko 2008; Ten Hoorn et al. 2016) but patients also have a right to communicate effectively, as is articulated in international documents such as the Universal Declaration of Human Rights (UDHR) (UN General Assembly 1948) and recently updated Patients’ Rights of the World Health Organization (WHO 2018). Besides, communication forms part of a nurse’s role of care (Finke et al. 2008). Communication is usually initiated by nurses and is restricted to task- and procedure-oriented messages, yes/no questions or directing the conversation to predictable answers (Patak et al. 2009; Radtke, Tate & Happ 2012). Not only are providing life-saving interventions and meeting critical intervention needs vital to the functioning of ICSs but also establishing adequate communication is essential. Any breakdown in communication between nursing staff and patients can have serious health repercussions, as is reflected in the following statement from a patient in the ICS who was temporarily not able to speak:

One night I was being given medication and had a food tube down my nose. I started regurgitating. It had come up out of my stomach. I was trying to make the nurse understand that I was regurgitating and for her to stop pumping anything more into my stomach. And it really got so bad that I ended up with a code 99. ‘No.’ I just kept shaking my head, No, don’t do that’…. (Fried-Oken, Howard & Stewart 1991:49)

Furthermore, the need to communicate vital information and to express basic physical needs (i.e. pain and discomfort, difficulty breathing, use of restraints and suctioning) is recognised by nurses managing the ICSs (Ten Hoorn et al. 2016; Wloszczak-Szubzda & Jarosz 2012). In a survey conducted with practising critical care nurses on the usage of various intervention techniques, Titler, Bulechek and McCloskey (1996) reported that communication enhancement was listed not only as one of the core behavioural interventions. It also received the same frequency of intervention score on a rating scale as several other medical interventions, such as fluid and pressure management, medication administration and infection control. Despite strong agreement on the issue of communication enhancement by critical care nurses, the paucity of empirically based communication assessment and intervention techniques can have serious medical consequences (Ten Hoorn et al. 2016).

Augmentative and alternative communication strategies can facilitate communication for patients in the ICS who are vulnerable communicators (Beukelman & Mirenda 2013; Grossbach et al. 2010; Happ et al. 2015; Radtke et al. 2012). Nursing staff generally are not adequately trained in augmentative and alternative communication techniques (Happ et al. 2015; Radtke et al. 2012). It is therefore essential to include the services of speech-language therapists in critical care settings to support and train nurses on how to provide communication-vulnerable patients access to augmentative and alternative communication strategies in the ICS (Blackstone 2015; Garrett et al. 2007; Happ et al. 2015; Hurtig et al. 2015; Radtke et al. 2012). Communication can be achieved through alternative strategies such as speaking valves, interpreting facial expressions, lip reading, gestures, communication boards, speech-generating devices, and writing or typing (Happ et al. 2011; Hurtig et al. 2015; McGrath et al. 2016). However, studies have shown that patients were significantly more satisfied when communicating with a paper-based communication board during mechanical ventilation, compared to patients who did not use a communication board at all (Happ et al. 2014). Thus, implementing augmentative and alternative communication strategies such as communication boards may pre-empt communication breakdowns between patients and nurses in the ICS. However, the effective implementation of augmentative and alternative communication strategies depends on whether nurses have received the necessary training, as well as whether they actually have access to resources such as communication boards.

According to Wloszczak-Szubzda and Jarosz (2012), communication problems were one of the challenges that nurses from Poland experienced because communication competences acquired during their undergraduate nursing education seem to regress during occupational activity. The authors therefore suggested updated communication skills training as a possible solution. Similarly, Hemsley, Balandin and Worrall (2012) highlighted the need for training nurses in the use of augmentative and alternative communication intervention strategies such as pen-and-paper communication boards in the ICS. The same recommendation was also made by Magnus and Turkington (2006) after a pilot study to determine patients’ and staff members’ experiences and perceptions of communication interaction in the ICS. In a systematic review on the use of various communication methods with mechanically ventilated patients in the ICS, Ten Hoorn and colleagues (2016) also underlined the importance of training ICS staff in the use of various augmentative and alternative communication intervention strategies to ensure implementation of such strategies. The implementation of such augmentative and alternative communication strategies could assist nurses to understand and appreciate the communication efforts by their patients, as the latter was one of the communication challenges experienced by patients in the ICS (Happ et al. 2014; Hurtig et al. 2015; Magnus & Turkington 2006). Addressing this communication challenge will potentially improve patient-provider care, and lower the stress levels for patients, their families and members of the health care staff (Happ et al. 2014; Hurtig et al. 2015; Ten Hoorn et al. 2016).

The main aim of this study was to compare the perspectives of nurses regarding communication with patients in an ICS by using an augmentative and alternative communication intervention strategy, namely the translated Setswana Vidatak EZ Board^TM^. Perspectives were measured before training, after a training session and after the communication board had been implemented for a 2-week period. Furthermore, the study aimed to expand ICS nurses’ knowledge and skills regarding communicating with patients in an ICS.

## Research methods and design

### Study design

A quasi-experimental pre-test post-test group design, from which participants were free to withdraw, as well as a control group, was used for a total of three measurement points.

### Setting

A 1650-bed public hospital with a 26-bed ICS (six beds reserved for cardiothoracic patients in the ICS and the rest to accommodate patients with other aetiologies) in a semi-urban, low socio-economic area in South Africa served as the research setting. This training hospital serves primarily Setswana-speaking patients.

### Study population and sampling strategy

Purposive sampling was used and nurses who met the following requirements were included: being a registered or enrolled nurse; working in the ICS for at least 3 months; and competent in spoken and written English and Setswana. Forty informed consent letters were distributed to potential participants, of whom 30 consented to participate. Of these 30 participants, all but one was female and their descriptive information is shown in Table 1.

**TABLE 1a:** Participant description (*N* = 30).

Variable	Experimental group (*n* = 15)	Control group (*n* = 15)
**Age**		
Range	24–57 years	25–60 years
Mean	36.5	49.9
s.d.	10.5	11.3
**Designation (%)**		
Registered nurse who specialises in critical care	33	60
Registered nurse with experience in critical care	60	33
Enrolled nurse	7	7
**Highest educational qualification (%)**		
Post-basic diploma in critical care nursing	33	47
Post graduate diploma in critical care nursing	7	13
Other (e.g. diploma in general nursing)	60	40
**Years’ experience working in ICS**		
Range	0.42–21 years	1–28 years
Mean	6.36	10.6
s.d.	7.66	9.34

ICS, intensive care setting; s.d., standard deviation.

**TABLE 1b:** Language proficiency.

Proficiency	Good (%)	Average (%)	Poor (%)	Good (%)	Average (%)	Poor (%)
Speak English	100	0	0	87	13	0
Read English	100	0	0	93	7	0
Write English	100	0	0	100	0	0
Speak Setswana	93	7	0	93	7	0
Read Setswana	87	0	13	93	7	0
Write Setswana	87	0	13	80	20	0

### Intervention materials

The intervention in this study entails the use of augmentative and alternative communication strategies implementing the Vidatak EZ Board™, a low-technology communication board that was specifically developed for patients in the ICS who experience communication difficulties (Patak et al. 2004, 2006). The Vidatak EZ Board™ contains alphabet letters, numbers (0–9), single words, word phrases (‘I want’ and ‘I am’), and an anterior and posterior picture of a genderless human body named ‘Pain Chart’. In addition, there is a vertical pain scale from 0 to 10. On the far right, space is provided where the patient can write if needed (Patak et al. 2006).

Following permission from the developer, the board was translated into Setswana because it is the official South African language spoken by the community in the region where the board was used (South Africa information 2012). A rigorous blind back-translation procedure was followed (Bornman et al. 2010; Peña 2007). Four translators, who were first language Setswana speakers and familiar with the culture, participated in the four-step translation process given as follows:

*Step 1*: Translator 1 translated the original English words on the Vidatak EZ Board™ from English to Setswana.

*Step 2*: Translators 2 and 3 (who were not familiar with the original English board) translated the Setswana words back to English.

*Step 3*: Translators 1, 2 and 3 met and reviewed the words on the English board and compared it with the Setswana words. Some English words had synonyms and the translators agreed upon better descriptive words for the Setswana translation.

*Step 4*: the three translators met with another translator (Translator 4) to discuss any disagreements until mutual agreement was reached for best descriptive Setswana words to reflect the original English words (Gropp 2015).

An example of the Setswana Vidatak EZ Board™ is shown in Online Appendix 1.

As this study employed a quasi-experimental pre-test post-test group design, the experimental group had to receive training to improve their knowledge on the implementation of the augmentative and alternative communication intervention strategies using the translated Vidatak EZ Board™. A detailed discussion on this training programme is as follows.

### Training of participants

The first author, a speech-language therapist, conducted the training using a PowerPoint presentation displayed on a laptop. A training programme was developed based on that of Radtke and colleagues (2012) to train the nurses in the experimental group. As per one of the conditions under which permission to conduct the training was obtained, the training had to be done within an hour because the ICS nurses could not be away from their patients for a longer period. Therefore, the training was conducted with one or two participants at a time. Exactly the same training format and information was shared in each training session as the presenter followed a script. The control group received neither the training nor the translated Vidatak EZ Boards™. The training programme aimed to enhance nurses’ knowledge and skills to communicate with patients in an ICS by focusing on care strategies through relationship building (Koloroutis 2004; Radtke et al. 2012). Definitions of communication, its importance in the ICS, what augmentative and alternative communication entails, and a demonstration on how to implement the communication board were covered in this 1-h training session. Strategies to improve communication between nurses and patients in the ICS were also addressed by highlighting the value of using the Vidatak EZ Board™.

### Data collection

#### Data collection instruments

Apart from the Vidatak EZ Board™ and the training programme that were used during the intervention and training as discussed earlier, a customised three-section questionnaire (Online Appendix 2) was developed based on surveys by Costello, Patak and Pritchard (2010), Hemsley et al. (2001) and Patak et al. (2009). Section A addressed the participants’ biographical information, while Section B focused on different communication aspects between nurses and patients, as well as possible communication barriers. Section C addressed questions regarding communication using the communication board. Section C was only completed after intervention and implemented as part of post-test 1. The questionnaire consisted of four closed-ended questions, 13 checklist items, six open-ended questions and five Likert scale options. Attempts were made to keep the questionnaire as short as possible because of the limited time that nurses in the ICS had to participate in this study.

#### Data collection procedures

Data collection commenced after ethics approval and permission from the relevant authorities. Participants were purposively assigned to either the experimental or control groups. The first 15 nurses who worked the day shift and consented to participate formed the experimental group and the first 15 nurses who worked the night shift and consented to participate constituted the control group.

The following procedures were followed for the experimental group: After a meeting with the unit manager, a time was scheduled to visit the ICS nurses during their tea break to explain the aim, duration and procedures of the study. This process was repeated on several days to recruit nurses from different day shift groups. Once ICS nurses confirmed their intent to participate, informed consent letters were distributed with the pre-test questionnaires. The first author negotiated a time and date for training with each participant individually. On the day of the scheduled training, signed consent forms and pre-test questionnaires were collected, followed by an hour-long training session on how to communicate with a patient using a translated communication board. Each nurse received a Setswana Vidatak EZ Board™ during training and had 2 weeks to implement the augmentative and alternative communication strategies with patients in the ICS. During this time, the researchers had no contact with the participants. Post-test 1 was distributed 2 weeks after training at the start of their shift, and the completed test was collected at the end of their shift on the same day. Following the same procedure, post-test 2 was completed 2 weeks after post-test 1.

For the control group, the procedures for recruitment and testing were identical to those for the experimental group, except that they did not receive training or the Vidatak EZ Board™ and only completed the pre-test and post-test 1 after 2 weeks.

#### Reliability and Validity

A rigorous blind-back translation procedure ensured the construct validity and cultural equivalence of the translated measure. Soliciting expert input confirmed face validity while test-retest reliability and stability was addressed through the use of the same questionnaire for the pre- and post-tests. The potential carry-over effect was acknowledged by allowing a time lapse of two weeks between the pre- and post-test measurements.

### Data analysis

This study used non-parametric statistics to analyse the ordinal data (Field 2013). These statistics are typically used with small groups and when the data do not follow parametric assumption. There were 15 participants in each of the experimental and control groups (*N* = 30), but some participants did not respond to all questions in the questionnaire – resulting in a varied number of data points across participants. Responses were divided according to subsections of the questionnaire. As a result, there were 56 different sets of responses to this study. A between-group Mann–Whitney *U* test was used to determine significant differences between the experimental and control groups on each of the 56 data sets. Specifically, data obtained for pre-test and post-test 1 were compared as the participants in the control group did not receive post-test 2. Additionally, the effect of training on the dependent variable was determined for the experimental group (within group comparison), and the non-parametric Friedman test was used across the pre-test, post-test 1 and post-test 2 measurements.

### Ethical considerations

This research was approved by the Research Ethics Committee of the University of Pretoria and has been conducted according to the Declaration of Helsinki Code of Ethics of the World Medical Association (2013).

## Results

### Between-group comparison

Data were compared for pre-test and post-test 1 measures between experimental and control groups. Table 2 depicts the significant results that were obtained for various items in the questionnaire using the independent sample Mann–Whitney *U* test. Results indicated that there were significant differences on two pre-test items in the patient-related communication barriers category. For both of these questions, responses were lower for the experimental group than for the control group. Additionally, there were significant differences on five post-test 1 items across three categories between the groups.

**TABLE 2:** Between-group comparisons on the items that were significantly different on the Mann–Whitney U test for the experimental group and the control group.

Items and categories	Test	Differences in mean ranks between experimental (E) and control (C) groups	*U* statistic	*p*
**Category: Frequency with which patient-related communication barriers occur**				
Patient’s speech is not understandable (dysarthria)	Pre-test	E < C	51.5	0.0431
Patient has a history of a stroke	Pre-test	E < C	56.5	0.0073
**Category: Nurses’ current means of communication with their patients in ICS**				
I use a communication board	Post-test	E > C	157.0	0.0176
I use sign language	Post-test	E < C	63.0	0.0170
**Category: Frequency with which nurses use other means of communication with patients in ICS**				
I use a communication device	Post-test	E > C	132.0	0.0310
I provide patient with hearing aids	Post-test	E < C	58.0	0.0318
**Category: Frequency with which nurses consider specific environmental factors to lead to communication barriers in the ICS**				
ICS has limited privacy	Post-test	E < C	49.5	0.0365

ICS, intensive care setting.

**FIGURE 1:**
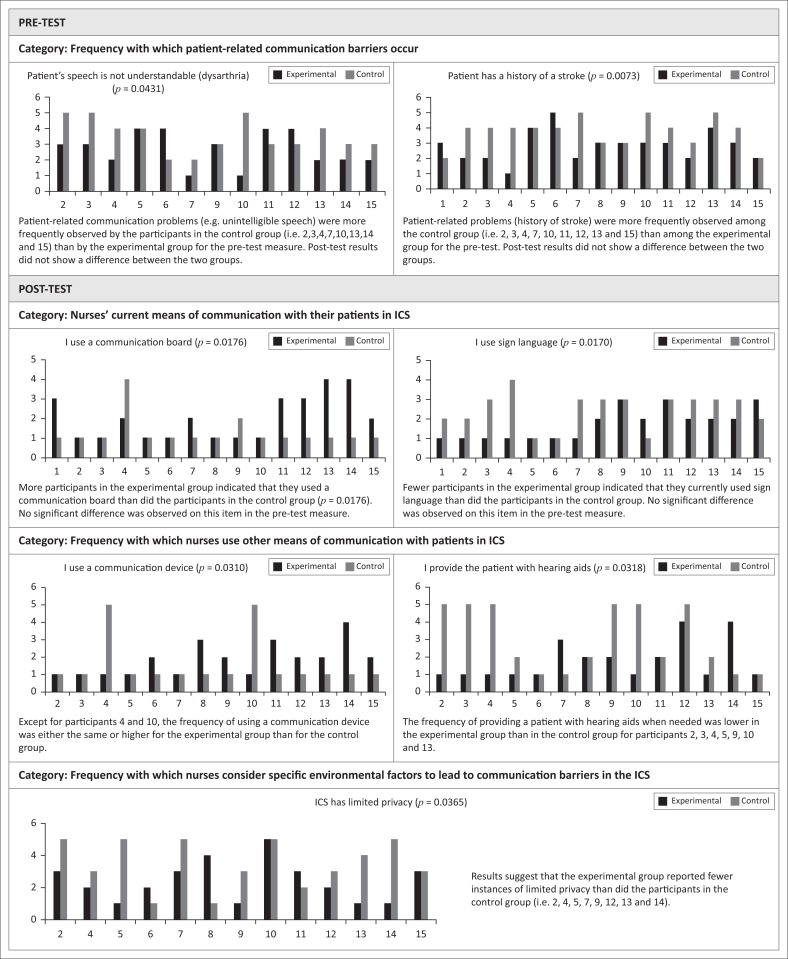
Individual participant responses in the pre-test and post-test for the experimental group and the control group. ICS, intensive care setting.

Regarding the question as to how nurses currently communicate with their patients in an ICS, results suggest that the participants in the experimental group used communication boards more frequently than those in the control group (*p* = 0.0176). This result is positive in the context that no such difference between groups was observed for that item on a pre-test measure. Furthermore, post-test results suggest that the experimental group used significantly fewer signs compared to the control group. Thus, it can be inferred that, as a result of the training, the communication board became a more frequent method of communication, while signs were less frequently used (*p* = 0.0170). There was no significant difference on the use of signs between the groups on the pre-test measure. Figure 1 presents the data on several items between groups and pre-test and post-test measures.

Regarding the frequency of using a particular method to communicate with patients: after training, the experimental group used both a communication board (*p* = 0.0176) and a communication device (*p* = 0.0310) more frequently for communication purposes.

The item *ICS has limited privacy* yielded statistically significant differences (*p* = 0.0365) between the control group and the experimental group on the post-test measure. Participants in the experimental group reported fewer instances of limited privacy than did participants in the control group. This can possibly be attributed to the positive effects of the training on concerns related to privacy and the trade-off between privacy and reducing communication barriers in the ICS.

### Within-group comparison in experimental group

To determine the effect of training, a Friedman two-way analysis of variance (ANOVA) was conducted for three measures (pre-test, post-test 1 and post-test 2). Significant results (*p* < 0.05) obtained from the Friedman test for five questionnaire items across three categories are shown in Table 3.

**TABLE 3:** Within-group comparison on the items that were significantly different on the Friedman test for the experimental group.

Items and categories	Statistic	*p*
**Category: Nurses’ current means of communication with their patients in ICS**		
I use a communication board	8.7200	0.0128
I use my mouth or lips	7.7857	0.0204
**Category: Frequency with which nurses use different communication modes with patients in ICS**		
I use a communication device	11.4375	0.0033
**Category: Frequency with which nurses consider specific nurse-related characteristics to result in communication barriers in the ICS**		
I am not easily available in the ICS	6.5000	0.0388
I have to focus on the health issues	7.1818	0.0276

ICS, intensive care setting.

**FIGURE 2:**
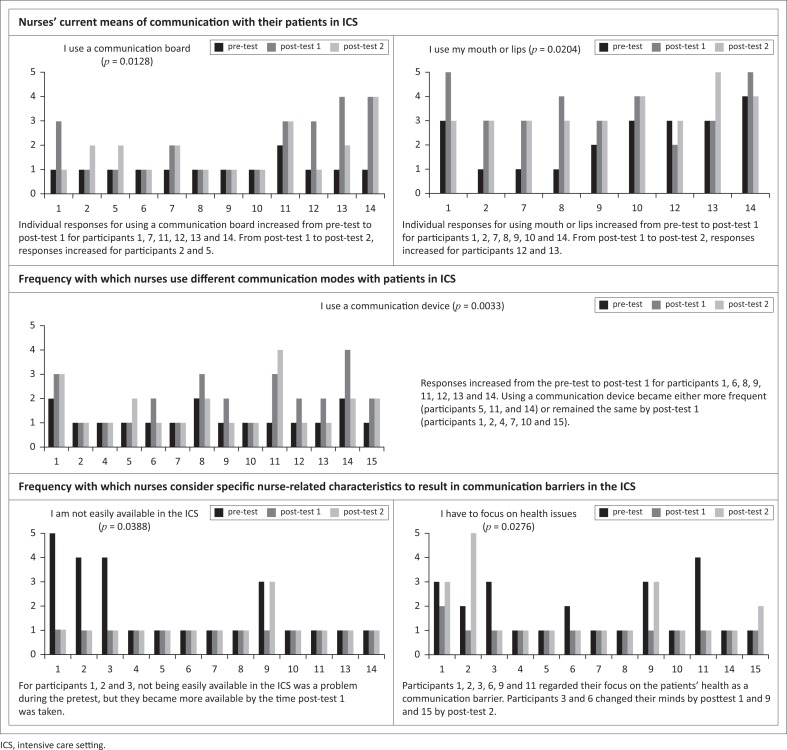
Individual participant responses for pre-test, post-test, and post-test 1 for experimental group.

Regarding the category on how nurses currently communicate with their patients in an ICS, results suggest that there was a significant difference in responses across three measures for two items, namely for the use of a communication board and for the use of mouth or lips. In Figure 2, the bar charts show that individual responses for these two items mostly increased from the pre-test to post-test 1 and that this increase continued from post-test 1 to post-test 2 for most participants. In summary, the training increased participants’ use of communication boards or use of mouth and lips to communicate with patients.

Unlike the between-group comparison, which did not show a statistically significant difference between any of the items for the category related to the frequency with which participants thought specific nurse-related characteristics resulted in communication barriers, two items yielded significant results within the experimental group, namely ‘I am not easily available in ICS’ (*p* = 0.0388) and ‘I have to focus on health issues’ (*p* = 0.0276). However, when the bar graphs of the individual responses in Figure 2 are examined, it seems that for most of the participants ‘I am not easily available in the ICS’ was not a factor that affected their communication with patients. Responses related to nurses focusing on health issues and nurse-related characteristics causing a communication barrier were similar to the responses regarding not being available in the ICS. Except for a few individuals, participants (even those who did not receive the training) considered communication to be as important as the patient’s health.

Apart from the quantitative results, some qualitative comments were made related to suggestion for specific adaptations to the communication board, for example to enlarge the font on the boards (‘make the written words bigger’) and decrease the number of written word options on the board (‘there are too many words on the board’; ‘let the patient only choose between a few words because they are very ill and will struggle to read through all the words’). Examples for one word options that were provided were ‘pain’, ‘uncomfortable’, ‘thirsty’ and ‘help’.

## Discussion

This study investigated nurses’ perspectives regarding the use of a communication board, specifically a translated Vidatak EZ Board^TM^, as an augmentative and alternative communication intervention strategy in the ICS following a brief training session. Although there is general consensus that communication is a vital component in the provision of appropriate patient care (Hemsley et al. 2012; Ten Hoorn et al. 2016), ICS nurses who care for intubated patients often indicate that they also experience frustration because of communication challenges (Grossbach et al. 2010). As such, the use of a communication board could assist nurses to understand patients’ communication efforts and obtain information from them on their needs and wants to ensure improved patient-provider care and patient outcomes, and lower stress levels for patients, their families and members of health care staff (Happ et al. 2014; Martinho & Rodrigues 2016; Patak et al. 2009; Ten Hoorn et al. 2016).

Despite a relatively small number of participants (*N* = 30) in this study, the results indicate that there were some changes in the perspectives of the participants after a short training session on the implementation of a communication board as an augmentative and alternative communication strategy in the ICS. However, their scores on post-test 2 (2 weeks after training) were lower than their post-test 1 scores, which might indicate that they did not implement the communication boards permanently and gradually stopped using them. Reasons for this could possibly be that the initial training on the implementation of the boards was too short to bring about permanent and sustainable change or that the generic nature of the training (as opposed to case-based training) was less effective in this specific context.

Another possible reason could be related to the training content. The training content focused on knowledge (e.g. increasing nurses’ understanding of the value of communication using communication boards) without focusing on the skills component (e.g. more hands-on practice opportunities) or the attitude component (Wloszczak-Szubzda & Jarosz 2012). When the researchers interacted with participants following the training, they were asked if the communication boards were available in English because they felt that the English boards would be more appropriate than the translated Setswana boards. A reason for this could be that English is typically the main language used officially and unofficially in health care settings in South Africa (Deumert 2010; Hussey 2012). The notion to prefer English might also reflect the extent to which globalisation has affected multilingual countries such as South Africa, resulting in English becoming the lingua franca (Khokhlova 2015). The position of English as an international language, its adoption by the liberation movements (especially in post-apartheid South Africa) and its widespread use in communication for administration, education and commerce, as well as the perception among many South Africans who speak an African language as their first language (such as the participants in the present study) about the desirability of speaking English (Khokhlova 2015), may have contributed to this phenomenon.

The short training that was conducted in this study certainly changed the initial behaviour of participants; however, the results were not sustained. Because communication skills are acquired through practice, follow-up training by means of additional practical exercises is suggested to reinforce the initial training on the use of the communication board (Wloszczak-Szubzda & Jarosz 2012). The nature of this training could possibly also be changed to focus on problem-solving skills in a case-based format (Wloszczak-Szubzda & Jarosz 2012).

For the patient-related category, there were differences (prior to the training) between the control group and the experimental group for two items: ‘Patient’s speech is not understandable’ (*p* = 0.0431) and ‘Patient has a history of a stroke’ (*p* = 0.0073). The control group was already of the opinion that these two items frequently lead to communication barriers. However, these differences disappeared after training, indicating that either the experimental group’s responses increased by post-test 1 to match those of the control group or that the responses of the control group decreased to match those of the experimental group. The difference between the two groups may be that the control group worked night shift and did not have to talk to the patients so often because the patients usually slept during the night shift.

## Strengths and limitations

The primary strength of this study is that most participants agreed that communication is critical to providing optimal health care in ICS settings. Specifically, preliminary data indicate potential for success in using a communication board in ICU settings. To limit knowledge transfer between the two groups and enhance internal validity, the participants who worked day shift were enrolled in the experimental group and those working night shift were allocated to the control group. The primary limitations were small sample size and lack of random assignments to experimental and control groups. Non-parametric statistics (typically used with small groups) were used to analyse the data, thus limiting the generalisability of the results.

## Recommendations for future research

It is suggested that future researchers consider investigating ICS patients’ and nurses’ perceptions on the contents of a communication board for use in ICSs in the South African context – this could be done either through focus groups or semi-structured or cognitive interviews with ICS nurses or critically ill participants who were admitted to ICSs and experience communication difficulties. Additionally, different types of training (e.g. case-based training spread over consecutive days using a problem-based learning focus) should be explored in an attempt to regulate the maintenance and generalisation of the communication board use after training. Further research on evaluating the perspective of nurses regarding the use of an English communication board with their patients will have clinical significance.

## Conclusion

This study is an attempt to provide preliminary empirical data on a communication tool in ICS. Despite a strong agreement on the issue of communication enhancement by critical care nurses, the lack of empirically based communication intervention strategies can lead to serious health repercussions. Participants agreed that communication is crucial in the ICS and that a communication board can be used successfully. However, only limited success was observed with the implementation of the board over time, possibly because of the brief training that was provided. This indicates that sustainable change is difficult to achieve with a short knowledge-based training session. It is therefore critical that the nurses and speech-language therapists work together to provide optimal health care to patients in ICS through the implementation of augmentative and alternative communication strategies.
